# COMP Report: CPQR technical quality control guidelines for conventional radiotherapy simulators

**DOI:** 10.1002/acm2.12356

**Published:** 2018-05-21

**Authors:** Marie‐Joëlle Bertrand

**Affiliations:** ^1^ Département de Radio‐Oncologie CIUSSS du Saguenay−Lac‐Saint‐Jean – Hôpital de Chicoutimi QC Canada

**Keywords:** conventional simulator, PCQR, quality control

## Abstract

The Canadian Organization of Medical Physicists (COMP), in close partnership with the Canadian Partnership for Quality Radiotherapy (CPQR) has developed a series of Technical Quality Control (TQC) guidelines for radiation treatment equipment. These guidelines outline the performance objectives that equipment should meet in order to ensure an acceptable level of radiation treatment quality. The TQC guidelines have been rigorously reviewed and field tested in a variety of Canadian radiation treatment facilities. The development process enables rapid review and update to keep the guidelines current with changes in technology (the most updated version of this guideline can be found on the CPQR website). This particular TQC details recommended quality control testing of conventional radiotherapy simulators.

## 1 INTRODUCTION

The Canadian Partnership for Quality Radiotherapy (CPQR) is an alliance amongst the three key national professional organizations involved in the delivery of radiation treatment in Canada: the Canadian Association of Radiation Oncology (CARO), the Canadian Organization of Medical Physicists (COMP), and the Canadian Association of Medical Radiation Technologists (CAMRT). Financial and strategic backing is provided by the federal government through the Canadian Partnership Against Cancer (CPAC), a national resource for advancing cancer prevention and treatment. The mandate of the CPQR is to support the universal availability of high quality and safe radiotherapy for all Canadians through system performance improvement and the development of consensus‐based guidelines and indicators to aid in radiation treatment program development and evaluation.

This document contains detailed performance objectives and safety criteria for *Conventional Radiotherapy Simulators*. Please refer to the overarching document *Technical Quality Control Guidelines for Canadian Radiation Treatment Centres* 1 for a programmatic overview of technical quality control, and a description of how the performance objectives and criteria listed in this document should be interpreted. The development of the individual TQC guidelines is spearheaded by expert reviewers and involves broad stakeholder input from the medical physics and radiation oncology community.^2^

All information contained in this document is intended to be used at the discretion of each individual center to help guide quality and safety program improvement. There are no legal standards supporting this document; specific federal or provincial regulations and license conditions take precedence over the content of this document.

## 2 SYSTEM DESCRIPTION

Simulators are essentially general radiography/fluoroscopy units with mechanical and optical capabilities extended to reproduce the geometric conditions of megavoltage radiation treatment machines. The mechanical and optical systems for simulators, therefore, shall duplicate those for linear accelerators and Cobalt teletherapy units, as appropriate. The radiation production systems, however, are very different for simulators and accelerators, the former being low dose and low energy imaging systems, the latter being high dose and high energy treatment systems.

Radiotherapy simulators have two roles in the preparation of patients for radiotherapy. The first is localization in which the high contrast and resolution achievable with kilovoltage x rays are used to guide the oncologist in the determination of the anatomical volumes to receive therapeutic radiation doses and those to be avoided. The information obtained during localization can be used as the input to two‐dimensional dose computation. The second role is that of true simulation. Beams, which may have been designed during a three‐dimensional treatment planning process, are set on the patient and the oncologist confirms that the geometric aspects of the treatment intent are being met.

Basic simulator design varies little across manufacturers. Detailed descriptions of the conventional radiotherapy simulators have been reported in the literature.^3^, ^4^, ^5^, ^6^, ^7^, ^8^, ^9^, ^10^, ^11^, ^12^, ^13^, ^14^, ^15^, ^16^ A rotatable gantry c‐arm is mounted on a stand. The source end of the c‐arm supports an x ray head consisting of a shielded x ray tube, field delineation, and collimation systems; the opposing end supports an image receptor and film cassette holder. The x ray head is translatable to enable different focus‐to‐axis distances (FAD). A treatment couch capable of translation, elevation, and full rotation on a turntable is used to position the patient. A control console is located in a shielded area adjacent to the simulator room. Some of the mechanical and optical systems may also be operated using controls inside the simulator room, for example, a hand pendant.

Traditionally, the image receptor used most often has been an image intensifier. A permanent record of the x ray image has been acquired either through digitally capturing the image as presented on the TV monitor connected to the camera viewing the output phosphor of the image intensifier, or through the use of film. More recently, large area flat panel detectors have become widely available and these are finding increasing use in radiotherapy simulation.

A major difference between conventional radiography and therapy simulation is the large distance (typically 120–170 cm) between the x ray focal spot and the image receptor. Since the simulator has geometric flexibility (to rotate around the patient), the image receptor is further away from the patient. Furthermore, simulation often requires beam‐patient geometries not normally used in conventional radiography/fluoroscopy, such as lateral or oblique views through large body thicknesses. These requirements result in higher skin exposures than would be encountered in diagnostic radiography. The requirement for geometric flexibility also limits the amount of shielding that can be attached to the x ray image intensifier and precludes restrictions on x ray beam size.

## 3 RELATED TECHNICAL QUALITY CONTROL GUIDELINES

In order to comprehensively assess conventional radiotherapy simulators performance, additional guideline tests, as outlined in related CPQR Technical Quality Control (TQC) guidelines must also be completed and documented, as applicable. Related TQC guidelines, available at cpqr.ca, include:

- Safety Systems
- Major Dosimetry Equipment

## 4 TEST TABLES

**Table:** Table 1 Daily quality control tests

Designator	Test	Performance	
		Tolerance	Action
Daily			
DS1	Collision avoidance	Functional	
DS2	Lasers/crosswires	1 mm	2 mm
DS3	Optical distance indicator	1 mm	2 mm
DS4	Crosswires/reticle/block tray	1 mm	2 mm
DS5	Light/radiation coincidence	1 mm	2 mm
DS6	Optical and x ray field size indicators	1 mm	2 mm

**Table:** Notes for daily tests

DS1	The configuration of this test will depend on the design of the facility and equipment. Safety is the concern and tests should be designed accordingly. As a minimum, manufacturer's recommendations and applicable regulations shall be followed.
DS2	Alignment of crosswires and appropriate lasers for collimator angle 0°, gantry angles 0°, 90°, and 270° at the isocenter.
DS3	Gantry angle 0° and at isocenter.
DS4	Coincidence of crosswires and/or reticle and/or block tray axes for collimator angle 0°, gantry angle 0° at isocenter.
DS5	Coincidence of the x ray and optical images of the field defining wires for a 10 × 10 cm^2^ field with a gantry angle 0°, collimator angle 0°, and source‐to‐surface distance (SSD) 100 cm. The tolerance and action levels apply to each field border. With an appropriate tool the test should be performed using the real time imaging device.
DS6	Both the optical and x ray images of the field defining wires for each field border should agree with the electronically indicated field size within the specified tolerance and action levels and for the geometry in DS5 above. With a verified reticle these tests can be performed with the aid of the real‐time imaging device.

**Table:** Table 2 Monthly quality control tests

Designator	Test	Performance	
		Tolerance	Action
Monthly			
MS1	Gantry angle readouts	1°	
MS2	Collimator angle readouts	1°	
MS3	Wires perpendicularity and orthogonality	1°	
MS4	Verification of FAD setting	1 mm	2 mm
MS5	Image amplifier movement	Functional	
MS6	Couch isocenter	1 mm	2 mm
MS7	Couch parallelism	1 mm	2 mm
MS8	Couch angle	1°	
MS9	Couch position readouts	1 mm	2 mm
MS10	Couch displacement	1 mm	2 mm
MS11	Laser/crosswire isocentricity	1 mm	2 mm
MS12	Optical distance indicator	1 mm	2 mm
MS13	Crosswire centring	1 mm	2 mm
MS14	Light/radiation coincidence	1 mm	2 mm
MS15	Field size indicators	1 mm	2 mm
MS16	Records	Complete

**Table:** Notes for monthly tests

MS1	Mechanical and digital gantry angle readouts shall be verified using a spirit level, or other appropriate levelling device, for at least 0°, 90°, 180°, and 270°.
MS2	After determination of the 0° collimator position, which is then used as a reference, mechanical and digital collimator angle readouts shall be verified using millimeter paper for at least 0°, 90°, and 270°.
MS3	This test refers to the field wires orthogonality and to their perpendicularity to the crosswires. This test should be performed on both the optical and radiation image.
MS4	Automatic setting of the focus‐axis‐distance shall be checked, if relevant, using mechanical devices.
MS5	The possibility to move the amplifier to limits (determined at commissioning) in three cardinal axes should be verified.
MS6	The couch isocentricity shall be checked over a range of couch angles from 90° to 270°. The tolerance and action levels refer to the maximum displacement of crosshair projection from the initial position in the isocenter plane.
MS7	With a couch angle 0°, couch motions shall be parallel the cardinal axes of the simulator geometry over an appropriate clinical range.
MS8	The couch rotation angle shall be verified over an appropriate clinical range. Deviation between the true 0° and the mechanical and numerical scale should be determined.
MS9	Mechanical and digital couch position readouts shall be verified over an appropriate clinical range in the directions of the three cardinal axes, if relevant.
MS10	Measurement of couch relative displacement in all three cardinal axes should be verified against digital readouts.
MS11	The radiation isocenter is established radiologically using the real time imaging device. Alignment of the crosswire and lasers at the isocenter is then confirmed for gantry angles of 0°, 90°, and 270°. The tolerance and action levels refer to deviation between the measured system and the isocenter.
MS12	A mechanical device, calibrated against the true radiation isocenter, is used to provide the base reading for the check of the optical distance indicator. The standards stated in Table 2 apply at the isocenter. The optical distance indicator should be checked over a clinically relevant range of SSD and gantry angle. The tolerance and action levels may be twice as large (i.e., 2 and 4 mm) at the clinical limits of the optical distance indicator's range.
MS13	The coincidence of both the optical and radiological images of the crosswires are measured with respect to radiological isocenter at 100 cm SSD for collimator angles of 0°, 90°, and 270°. The tolerance and action levels refer to the coincidence with the radiation isocenter.
MS14	Geometric alignment of the x ray and optical images of the field defining wires shall be established over a range of field sizes from 5 × 5 cm^2^ to 35 × 35 cm^2^ at gantry angles 0°, 90°, and 270°. Representative half‐blocked fields shall be included. A minimum of six field sizes will be required for this test. The tolerance and action levels apply to each edge of a rectangular field.
MS15	Compliance of the x ray and optical images of the field defining wires with the indicated dimensions shall be established over a range of field sizes from 5 × 5 cm^2^ to 35 × 35 cm^2^ at gantry angles 0°, 90°, and 270°. Representative half‐blocked fields shall be included. A minimum of six field sizes will be required for this test. Different field sizes should be examined at different gantry angles if appropriate and efficient. The tolerance and action levels apply to each edge of a rectangular field.
MS16	Documentation relating to the daily quality control checks, preventive maintenance, service calls, and subsequent checks shall be complete, legible, and the operator identified.

**Table:** Table 3 Semiannual quality control tests

Designator	Test	Performance	
		Tolerance	Action
Semiannually			
SS1	Lead apron	Functional	
SS2	Focal spot	Reproducible	
SS3	Contrast	Reproducible	
SS4	Resolution	Reproducible	
SS5	Fluoroscopic timer	5%	10%

**Table:** Notes for semiannually tests

SS1	Any available lead aprons, gloves, and other protective wear should be visually and radiologically inspected for cracks and appropriate action taken should cracks be found.
SS2−4	A variety of equipment is available for performing these tests. The tolerance and action levels will need to be developed locally depending on the equipment available and the performance variability in the observers. Routine monitoring of these parameters should be based on performance at installation.
SS5	The limit on fluoroscopy time is verified.

**Table:** Table 4 Annual quality control tests

Designator	Test	Performance	
		Tolerance	Action
Annually			
AS1	Isocenter definition and coincidence	1 mm	2 mm
AS2	Crosswire centring	1 mm	2 mm
AS3	Couch deflection	3 mm	5 mm
AS4	Alignment of focal spots	0.5 mm	1 mm
AS5	kVp	5%	10%
AS6	Reference dosimetry	5%	10%
AS7	Beam quality (half‐value layer)	5%	10%
AS8	Automatic exposure control	5%	10%
AS9	Independent quality control review	Complete

**Table:** Notes for Annual tests

AS1	The mechanical, optical, and radiation isocenter should be redefined and optical and mechanical systems realigned. Coincidence between gantry, collimator, and couch isocenters shall be verified.
AS2	Crosswire centring with gantry at 0° and at least 2 different source‐axis distances (SADs).
AS3	Couch deflection is measured with 70 kg at the end with the couch extended to the isocenter.
AS4	Typical exposure factors are used.
AS5	kVp should be measured at least three settings over the range from 60−120 kVp. When measured non‐invasively, tolerances and action levels should refer to baseline values established at acceptance and referenced to invasive measurements.
AS6	Tolerance and action levels refer to the coefficient of variation in 10 measurements of relative exposure at a typical set of operating parameters. These tests should be performed with and without automatic exposure control.
AS7	Half‐value layer (HVL) is to be compared at three kVp values with the baseline values established at acceptance.
AS8	Where more than one detector can be used for automatic exposure control, consistency between the exposures delivered should be established.
AS9	To ensure redundancy and adequate monitoring, a second qualified medical physicist shall independently verify the implementation, analysis, and interpretation of the quality control tests at least annually.

## CONFLICT OF INTEREST

The author has no conflict of interest to disclose.
